# Characterizing the gut microbiota composition in Taiwanese hypertensive patients using 16S rRNA sequencing analysis

**DOI:** 10.7150/ijms.109340

**Published:** 2025-04-28

**Authors:** Ming-Shan Chen, Shin-Kuang Jiang, Zhi-Yong Chong, Jou-Wei Chiang, Yan-Min Chen, Hsin-Yu Huang, Jui-Chieh Chen

**Affiliations:** 1 Department of Anesthesiology, Ditmanson Medical Foundation Chia-Yi Christian Hospital, Chiayi city 60002, Taiwan; 2 Department of Medical Laboratory Science and Biotechnology, Asia University, Taichung City 41354, Taiwan.; 3 Department of Neurology, China Medical University Hospital, China Medical University, Taichung City 404337, Taiwan.; 4 Department of Biochemical Science and Technology, National Chiayi University, Chiayi 600355, Taiwan.

**Keywords:** hypertension, gut microbiota, 16S-rRNA sequencing, taxonomic biomarkers, cardiocerebrovascular diseases

## Abstract

Hypertension (HTN) is a significant risk factor for cardiovascular and cerebrovascular diseases. Accumulating evidence suggests a close relationship between HTN and alterations in the gut microbiota composition and abundance.

We recruited 23 HTN patients and 17 controls matched for demographic characteristics. DNA extracted from fecal samples of patients was subjected to Illumina MiSeq sequencing, targeting the V3-V4 region of the bacterial 16S rRNA gene for analysis. We compared the diversity and composition of gut microbiota between the two groups. The α-diversity of gut microbiota in HTN patients was similar to that in the control group. β-diversity analysis showed slight differences in microbial composition between the HTN and control groups. We used Welch's *t*-test to evaluate the significant difference in the bacterial composition of the top 20 ASVs between the HTN group and the control group, and the results showed that *Tyzzerella* was significantly increased, while *Faecalibacterium* was significantly decreased in the HTN group. We performed PCR using *Faecalibacterium*-specific primers and analyzed their levels through agarose gel electrophoresis, confirming the reduced abundance of *Faecalibacterium* in the HTN group. In addition, Tax4Fun2 analysis was employed to examine differences in microbial functionality between the HTN group and the control group. In conclusion, we studied the fecal microbiota of HTN population in Taiwan through 16S rRNA gene sequencing, and found that *Faecalibacterium* has a lower abundance in HTN patients. This unique alteration in gut microbiota may provide insights into the pathogenesis of HTN and aid in the development of novel biomarkers and therapeutic targets.

## Introduction

Hypertension (HTN) is the primary risk factor for cardiovascular diseases. It has emerged as a major global public health issue, particularly affecting the elderly population, with a notable impact on women 1. Apart from age, detrimental dietary habits 2, endocrine imbalances 3, and genetic inheritance 4 have all been confirmed to contribute to the development of HTN.

The gut microbiota is a diverse community of microorganisms, including bacteria, fungi, and viruses, residing in the human intestinal tract. They form a symbiotic relationship with the human body and play a crucial role in regulating various physiological functions 5. In-depth exploration of the composition of the gut microbiota in various human diseases and the investigation of its symbiotic/dysbiotic mechanisms are crucial for their potential application in clinical disease treatment 6. In recent years, a growing body of evidence has shown a significant correlation between alterations in the gut microbiota composition and abundance and the development of HTN 7, 8. Recent research evidence has shown that probiotics play a key role in regulating blood pressure, making them a viable option for the prevention and treatment of HTN 9.

In recent years, the rapid advancement of sequencing technology has allowed us to further explore the relationship between genes and diseases. The occurrence of HTN has been confirmed to have a direct causal relationship with genetic variations at specific loci 10. However, the etiology of HTN is complex and heterogeneous, making it challenging to elucidate the pathogenic mechanisms solely based on human genetics. In the present study, we employed high-throughput sequencing of the 16S rRNA gene to explore the composition and diversity of the gut microbiota in HTN patients. Our aim is to identify potential bacterial biomarkers and provide novel insights and innovative treatment strategies for HTN.

## Materials and methods

### Participants

We enrolled hospitalized patients over the age of 60 in this prospective cohort study conducted at Ditmanson Medical Foundation Chia-Yi Christian Hospital in Chiayi City, Taiwan. The type of surgery was not restricted. Participants were divided into two groups: a total of 23 HTN patients and 17 normotensive controls were recruited. After obtaining approval from the Ethical Committee of Ditmanson Medical Foundation Chia-Yi Christian Hospital, the study was conducted. Clinical specimen collection and related research were carried out in accordance with the Institutional Review Board (IRB) agreement of the participating institution. The researchers recorded general information about the study subjects, including age, gender, height, weight, and blood pressure, etc. The diagnosis of HTN and normotensive status in this study was based on the 2022 Taiwan Hypertension Treatment Guidelines. Additionally, patients with HTN were those who had been on long-term antihypertensive medication. The inclusion criteria for both the HTN and control groups were patients aged over 60 years. The exclusion criteria for both groups included dementia, stroke, long-term bedridden status, and substance, alcohol, or drug abuse. To minimize hospitalization-related confounders, fecal samples were collected within 24 hours of admission, before any surgical procedures or major interventions. Patients who had received antibiotics within the past three months or during hospitalization before sample collection were excluded to control for microbiota alterations.

### Sample collection and DNA extraction

Approximately 1 g of feces samples was immediately transported to the laboratory after collection. The samples were stored at -80°C until further analysis. The genomic DNA of the feces was extracted using the QIAamp DNA Stool Mini Kit (Qiagen, Hilden, Germany) following the manufacturer's guidelines.

### 16S rDNA amplicon sequencing

The next-generation sequencing library preparation was performed using established primers and Illumina adapters targeting the V3-V4 region of the 16S rDNA. The V3-V4 regions of 16S rRNA genes were amplified using a specific primer (341F: 5′-CCTACGGGNGGCWGCAG-3′ and 805R: 5′-GACTACHVGGGTATCTAATCC-3′) with a barcode. After 16S sequencing on the Illumina MiSeq platform, the read data underwent quality control using FastQC and MultiQC to assess read quality. DADA2 was employed for filtering noisy sequences, error correction, chimeric sequence removal, and singleton filtering. Amplicon Sequence Variants (ASVs) were inferred using DADA2, which ensures accurate variants with low false positives and high sensitivity. Finally, the ASVs were classified using QIIME2.

### Diversity analysis

Alpha diversity analysis evaluates microbial diversity within individual samples using four diversity indices: Shannon, Simpson, observed_features, and Chao1. Beta diversity analysis assesses diversity between samples through principal component analysis (PCA), principal coordinates analysis (PCoA), and non-metric multidimensional scaling (NMDS).

### Species composition analysis

The stacked taxonomy level plot presents the sequence count information for different taxonomic levels (kingdom, phylum, class, order, family, genus, species) within each group. It displays the top ten most abundant species at the family level in each group. The differential abundance analysis includes three methods: (1) Comparing the significant bacteria between the two groups at each taxonomic level. (2) Selecting the top 20 ASVs with significantly different expression based on differential average values for comparison between the two groups. (3) Using Lefse to filter species with significant differences between the two groups based on the effect size, as determined by Linear discriminant analysis (LDA).

### Fecal detection of *Faecalibacterium genus* using PCR

PCR amplification of the *Faecalibacterium genus* 16S rRNA gene was performed using 10 ng/μl DNA samples and specific primers (Fprau223F 5′-GATGGCCTCGCGTCCGATTAG-3′, Fprau420R 5′-CCGAAGACCTTCTTCCTCC-3′). The PCR reaction involved initial denaturation at 95°C for 5 min, followed by 40 cycles of denaturation at 95°C for 30 s, primer annealing at 54°C for 30 s, extension at 72°C for 30 s, and a final extension at 72°C for 5 min. After PCR, the amplification products were analyzed by agarose gel electrophoresis using a 2% agarose gel. The DNA bands corresponding to the *Faecalibacterium genus* were visualized under UV light, and quantification was performed using ImageJ software. The band intensities were normalized to an internal reference gene to obtain relative quantification data for the *Faecalibacterium genus*.

### Predicting the potential functions and pathways involved in the composition of gut microbiota

Tax4Fun2 (v1.1.5) is a tool that predicts the functional composition of prokaryotic communities based on 16S rRNA gene sequences. In this study, we chose the NCBI RefSeq database as the reference for mapping the ASVs to 16S rRNA sequences using BLAST (v2.9.0). The abundance information was then converted into functional composition, and the results were presented in terms of KEGG metabolic pathways.

### Statistics

Differences in gut microbiota among HTN group and control group were assessed using Adonis. Means between two groups were compared using Mann-Whitney U test and Welch's t-test. For Table 1, age was compared using an independent samples t-test, while BMI was analyzed with Welch's t-test. Categorical variables (sex, DM, HLP, PUD, smoking, drinking, cancer, and BPH) were analyzed using chi-square tests or Fisher's exact tests, as appropriate. Valuable insights into the role of gut microbiota in HTN were provided by these methods.

## Results

### Population statistics and clinical characteristics of enrolled participants

This study recruited 40 participants, divided into two groups: the HTN group (*n* = 23) and the control group (*n* = 17). The mean age of the HTN group was 71.57 ± 1.538 years, comprising 17 men (73.9%) and 6 women (26.1%). The control group had a mean age of 72.59 ± 1.348 years, with 13 men (76.5%) and 4 women (23.5%). There were no significant differences between the two groups in terms of age (*p* = 0.653) or sex distribution (*p* = 0.935). However, the HTN group had a significantly higher body mass index (BMI) than the control group (26.19 ± 0.7438 kg/m² vs. 22.75 ± 0.8170 kg/m², *p* = 0.018). Regarding comorbidities, the prevalence of diabetes mellitus (DM) was significantly higher in the HTN group compared to the control group (43.5% vs. 17.6%, *p* = 0.035), as was the prevalence of hyperlipidemia (HLP) (39.1% vs. 5.9%, *p* = 0.002). Additionally, the control group had significantly higher proportions of peptic ulcer disease (PUD) and alcohol consumption compared to the HTN group (PUD: 23.5% vs. 4.3%, *p* = 0.043; alcohol consumption: 23.5% vs. 4.3%, *p* = 0.043). There were no significant differences between the two groups in terms of smoking, cancer, or benign prostatic hyperplasia (BPH) (*p* > 0.05). The detailed demographics and clinical characteristics of the HTN patients and control group are presented in Table 1.

### Sequencing data and taxonomy analysis for both groups

In total, 3,493,411 reads were obtained for all samples. The HTN group had an average of 88,434 reads per sample (range: 59,123 to 115,745), while the control group had an average of 85,849 reads per sample (range: 58,170 to 112,512). Analysis of shared and unique ASVs between the two groups was visualized using a Venn diagram. There were 284 ASVs that were shared between the two groups. However, the HTN group and control group exhibited 159 and 70 unique ASVs, respectively (Supplementary Figure 1A).

### Comparison of gut microbiome diversity and structure between groups

To further investigate the diversity and richness of the gut microbiota between the two groups, four indices were employed to assess α diversity, including community diversity (Shannon, Simpson) and community richness (observed species, Chao1). The Shannon and Simpson indices for the HTN group and the control group were 5.10 and 0.94, and 5.04 and 0.94, respectively (Supplementary Figure 1B-1C). The observed features and Chao1 indices for the HTN group and the control group were 103.17 and 103.17, and 97.53 and 97.59, respectively (Supplementary Figure 1D-1E). The results indicated that although there was no significant difference in α diversity between the HTN group and the control group (*p* > 0.05), the diversity and richness of gut microbiota appear slightly higher in the HTN group. To explore the differences in the structure and composition of gut microbiota between the two groups, we employed the β-diversity index (PCA, PCoA, and NMDS) for evaluation. The results revealed distinct clustering patterns in the PCA plot (PC1: 7.52%, PC2: 7.33%) (Supplementary Figure 1F), PCoA plot (PCoA1: 14%, PCoA2: 8%) (Supplementary Figure 1G), and NMDS plot (stress = 0.211) (Supplementary Figure 1H). The Adonis method was used for statistical testing, and no significant differences were found between the two groups.

### The relative bacterial abundances of gut microbiota in the two groups

**Figure 1:**
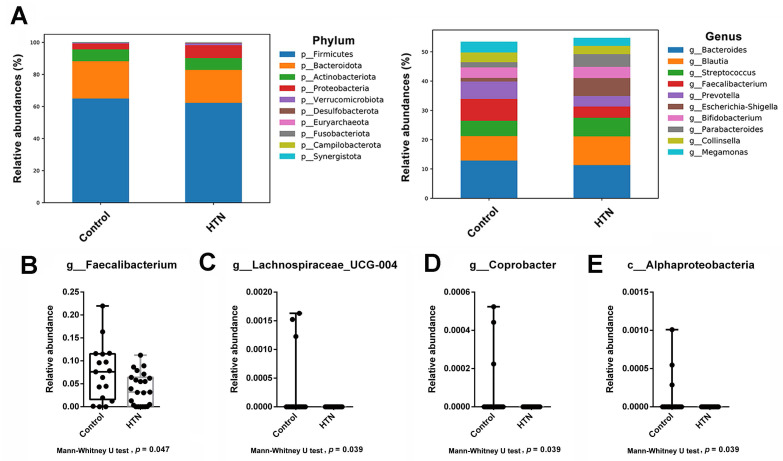
** The relative abundance of the most abundant taxa at various taxonomic levels (phylum, class, order, family, genus, and species) between the HTN and control groups.** (A) Top 10 taxa with the highest relative abundance of ASV at different taxonomic levels were compared between the two groups. At each taxonomic level, different colors were used to represent the distribution and proportion of distinct microbial taxa. (B-E) The box plot illustrates the Mann-Whitney U test comparing the relative abundances of each taxonomic unit with significant differences between the two groups. The bacteria with significant differences between the two groups included: (B) *g_Faecalibacterium* (C) *g_Lachnospiraceae_UCG-004*, (D) *g_Coprobacter*, and (E) *c_Alphaproteobacteria*. The y-axis represents the relative abundance, while the x-axis represents the different taxa at specific taxonomic levels. Boxplots display the median (center line), interquartile range (box), and whiskers representing the minimum and maximum values within 1.5 times the interquartile range. Outliers are represented as individual data points.

**Figure 2:**
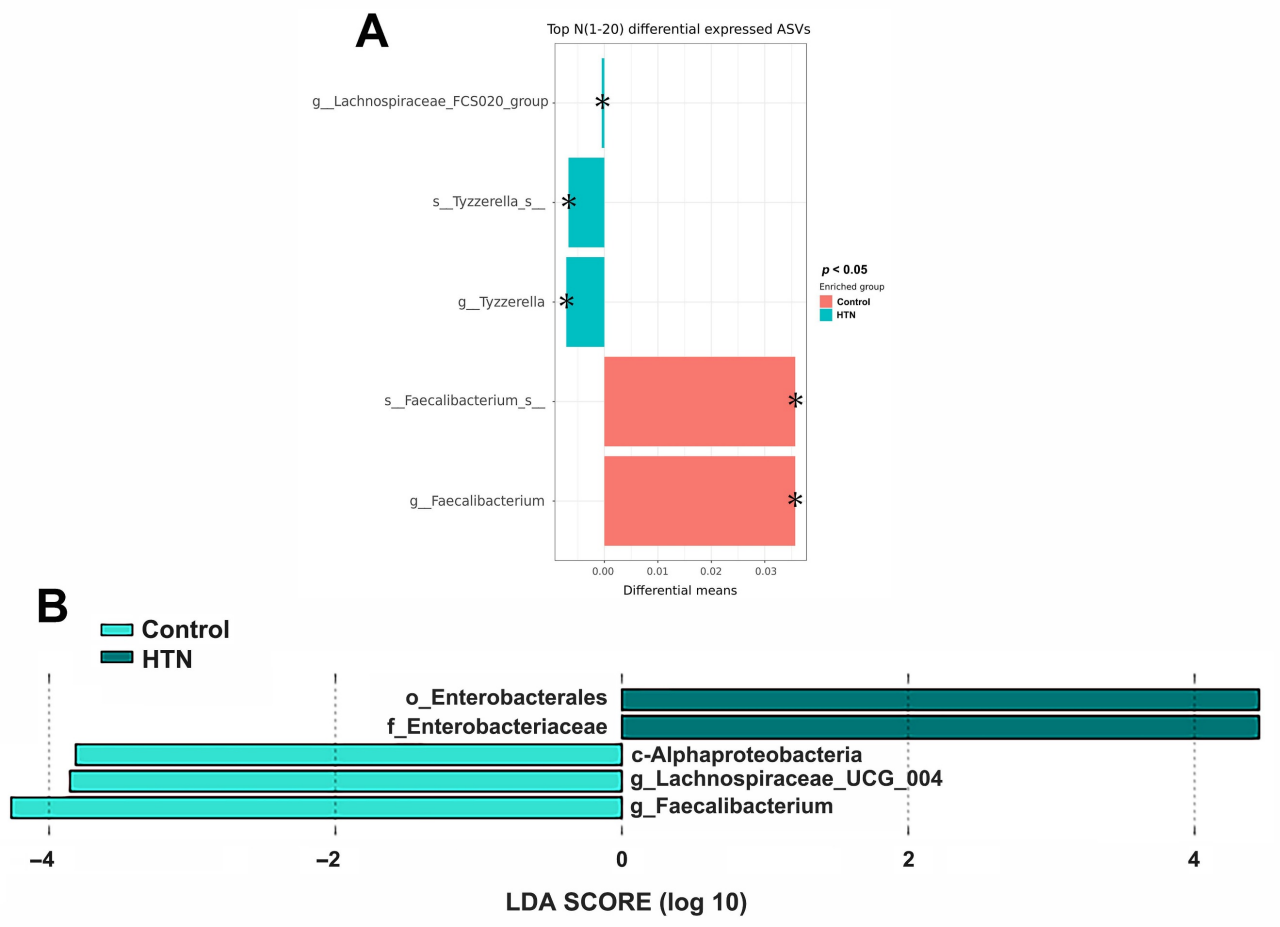
** Comparison of gut microbiota abundance between the HTN and control groups by Welch's t-test and LEfSe** (A) Differential abundances of the top 20 ASVs between the HTN and control groups. Pair-wise Welch's t-tests were performed to identify statistically significant differences in ASV abundances. The X-axis represents the absolute differential means value, while the y-axis displays the taxa names, ranging from species to higher taxonomic ranks. The asterisk (*) indicates statistically significant differences based on the *p*-value. (B) LEfSe analysis for identification of fecal microbiome members enriched in HTN or control groups. The bar plot represents the effect size (LDA score) of significant differential taxa identified using LEfSe analysis. The length of the bar corresponds to a log10 transformed LDA score, indicating the magnitude of the effect. The colors of the bars indicate the group: dark green represents HTN, and light green represents the control group. The histogram of LDA scores highlights the biomarkers with statistical differences between the HTN and control groups, with the length of the bars reflecting their respective influence levels.

We analyzed the taxonomic abundance differences between HTN patients and control subjects. As shown in Figure 1A and Supplementary Figure 2, the taxonomic compositional differences between the two groups were evaluated at six different classification levels: phylum, class, order, family, genus, and species. At each level of classification, we summarized the top 10 taxa with the highest relative abundances. In addition to analyzing the relative abundances of the top 10 taxa at six different classification levels between the two groups, we also employed the Mann-Whitney U test to identify bacteria that exhibited significant differences between HTN patients and control subjects. The results revealed a significant decrease in the abundance of *Alphaproteobacteria* at the class level in the gut of HTN patients. At the genus level, compared to the control group, HTN patients had significantly lower frequencies of *Faecalibacteria*, *Lachnospiraceae_UCG-004*, and *Coprobacter* in their gut (Figure 1B). Furthermore, we also utilized the top 20 differentially expressed ASVs and compared their relative abundances between the two groups. After conducting the Welch's *t*-test, the results indicated that the ASV abundances of *Lachnospiraceae_FCS020_group* and *Tyzzerella* were significantly increased in HTN patients, while the ASV abundance of *Faecalibacterium* was significantly decreased (Figure 2A). We then further analyzed using the LEfSe method and identified microbiota features that exhibited significant and biologically meaningful differences between the two groups based on LDA scores. As illustrated in Figure 2B, the HTN group and control group were enriched with 2 and 3 bacterial taxa, respectively. In the HTN group, *Enterobacterales* were found to be significantly enriched at the order level, and* Enterobacteriaceae* were significantly enriched at the family level. In contrast, in the control group, *Alphaproteobacteria* were significantly enriched at the class level, and *Lachnospiraceae_UCG_004* and *Faecalibacterium* were significantly enriched at the genus level. These findings provide valuable insights into the distinctive microbial characteristics associated with HTN and the control group.

### The relative quantification of *Faecalibacterium* genus in each fecal sample DNA from the HTN and control groups

**Figure 3:**
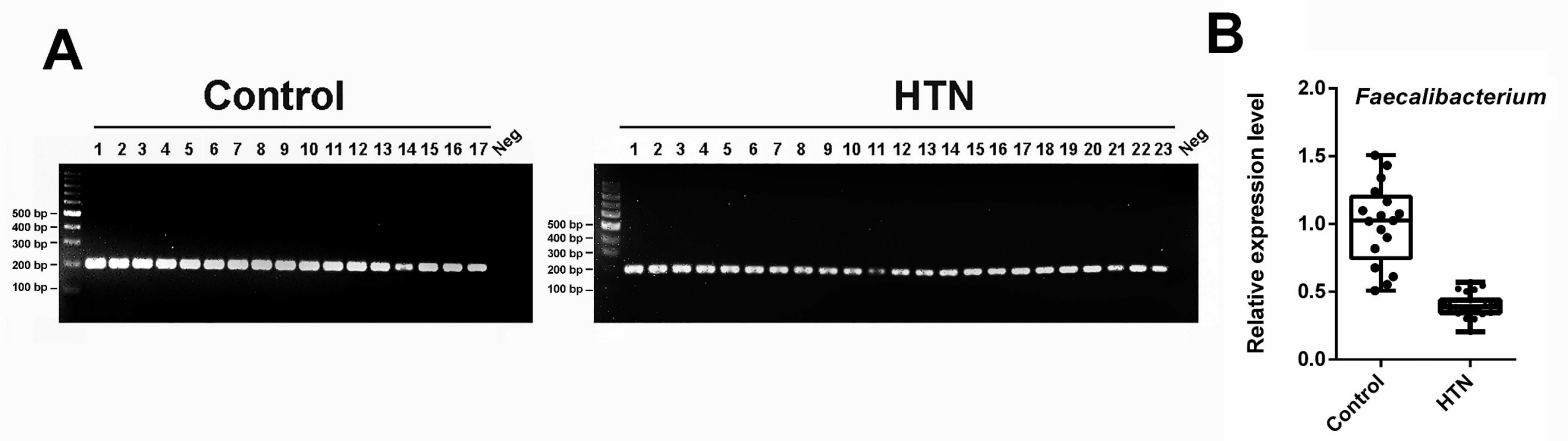
** The relative abundance of the genus *Faecalibacterium* in the HTN and control groups was quantified using species-specific PCR and agarose gel electrophoresis.** (A) PCR and agarose gel electrophoresis of fecal DNA from two groups of participants using 16S primers (Fprau223F/Fprau420R). M, DNA marker; panel left: lanes 1-17, DNA of control group; panel right: lanes 1-23, DNA of HTN group; N, negative control. (B) The PCR bands were quantified and presented using a box plot.

Based on all the aforementioned results, it is consistently shown that the abundance of *Faecalibacterium* is lower in the gut of HTN patients compared to the control group. Consequently, we proceeded with PCR amplification of each sample using the 16S primer pair Fprau223F/Fprau420R, followed by agarose gel electrophoresis to analyze the PCR products. As depicted in Figure 3A, only a single band was detected, and its intensity was lower in the HTN group compared to the control group. Further quantification of the electrophoresis bands, as presented in Figure 3B, revealed a significant reduction in the *Faecalibacterium* genus in the HTN group compared to the normotensive group.

### Comparative analysis of functional composition and KEGG pathway profiles in gut microbiota between HTN and control groups using Tax4Fun2

**Figure 4:**
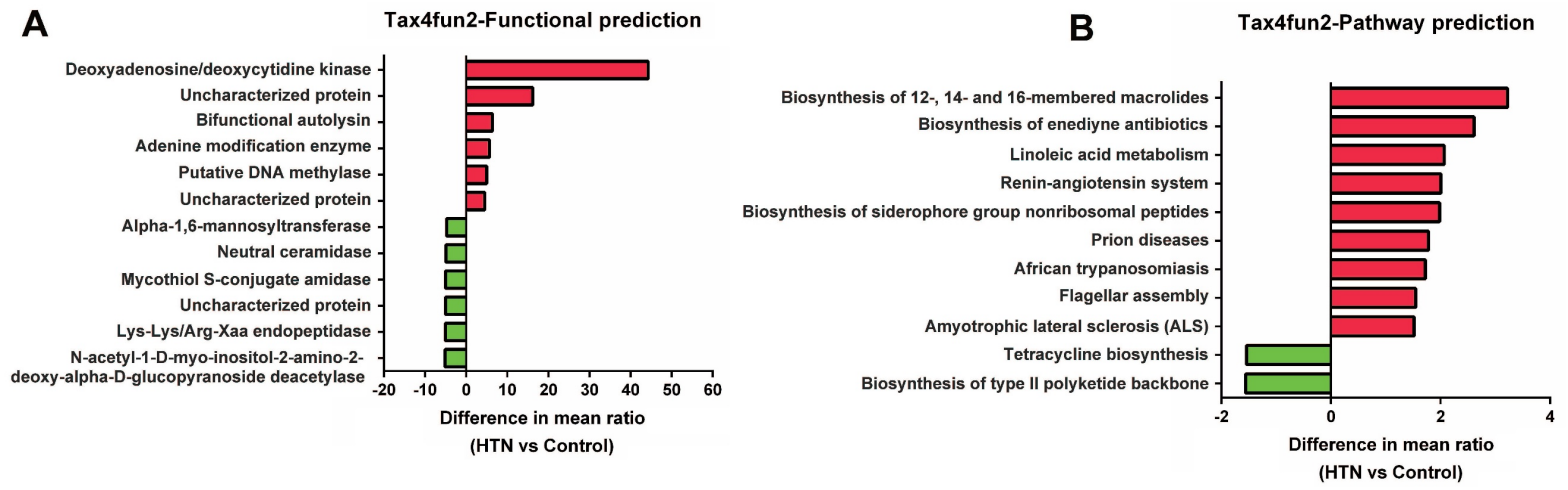
** Predicting functional composition and KEGG pathway differences in prokaryotic microbiome between HTN and control groups using Tax4Fun2.** (A) Analysis of potential functional differences between the two groups. (B) The correlation in KEGG pathway abundances was investigated.

To further understand the potential impact of these gut microbial communities on human health, we have used Tax4Fun2 to predict the functional composition and differences in KEGG pathways within the prokaryotic microbiome. The functional prediction results (Figure 4A) have shown that the relative abundance of adenine deaminase and DNA methyltransferase is significantly higher in the HTN group, while that of neutral ceramidase and Lys-Lys/Arg-Xaa endopeptidase is lower. Additionally, KEGG pathway analysis (Figure 4B) has indicated that the HTN group exhibits a higher relative abundance in functions related to linoleic acid metabolism and the renin-angiotensin system, whereas the relative abundance of pathways associated with tetracycline biosynthesis is lower. These functional and pathway alterations align with the observed changes in specific bacterial genera, such as the decreased relative abundance of *Faecalibacterium prausnitzii* and *Lachnospiraceae_UCG-004* and the increased abundance of *Tyzzerella* in the HTN group. These findings have provided valuable insights into the potential functional differences between the gut microbiota of the HTN and control groups.

## Discussion

The development of HTN is a complex, multifactorial process involving both genetic and environmental risk factors. With the decreasing cost of sequencing, an increasing number of studies are highlighting the correlation between the gut microbiota and blood pressure or HTN 11. In a previous study, the transplantation of cecal contents from HTN rats to normotensive rats induced the development of HTN 12, suggesting that the composition of the gut microbiota and its derived metabolites can regulate blood pressure.

Changes in the gut microbiota can lead to variations in microbial metabolites, subsequently exerting diverse effects on blood pressure regulation 13. Among these, short-chain fatty acids (SCFAs) most commonly refer to straight-chain species with 2-4 carbon atoms, such as acetate, propionate, and butyrate. Research has indicated that blood pressure fluctuations are consistently associated with changes in SCFAs 14. Additionally, studies using mouse models have demonstrated that administering propionate can lead to better control of HTN and reduce cardiovascular damage 15.

*Faecalibacterium prausnitzii* is considered a probiotic, believed to have significant physiological functions within the intestine. One key function is providing energy through the production of butyrate, which helps maintain intestinal barrier function and reduces inflammation 16. Additionally, the abundance and capacity of butyrate-producing bacteria in the gut are also associated with blood pressure 17. Previous studies have found that the abundance of *Faecalibacterium prausnitzii* is lower in individuals with HTN compared to those with normal blood pressure 18, 19. This result aligns with our research findings and suggests that *Faecalibacterium prausnitzii* may play a potentially influential role in the development of HTN.

In our experimental results, we found that the relative abundance of *Lachnospiraceae_UCG-004* in the gut of HTN patients was lower compared to the control group. *Lachnospiraceae* is an important group of gut bacteria known to produce butyrate, which contributes to maintaining gut health. In a study, it was indicated that *Lachnospiraceae* may alleviate stress-induced gastrointestinal symptoms, suggesting anti-inflammatory properties 20. Since HTN is a significant risk factor for stroke, other research reports have also indicated a reduction in *Lachnospiraceae* in stroke patients 21. Therefore, the decrease in butyrate-producing metabolites, leading to reduced butyrate concentrations, may contribute to the occurrence of various diseases, such as HTN and stroke. As for the dietary impact on gut microbiota composition, previous research has indicated that feeding mice a high-salt diet seems to lead to an increase in *Lachnospiraceae FCS020* 22. Given the close relationship between a high-salt diet and HTN, our LEfSe analysis results showed a significant increase in *Lachnospiraceae FCS020* in HTN patients. This raises the question of whether dietary influences on microbial distribution may contribute to the likelihood of developing HTN.

Our study found a significant reduction in *Faecalibacterium* and *Lachnospiraceae_UCG-004* in elderly HTN patients, which is consistent with the known gut microbiota characteristics in the elderly population. Literature suggests that these taxa are typically reduced in older adults receiving long-term care and are associated with frailty and inflammation—both of which are linked to an increased risk of HTN 23. Additionally, growing evidence indicates that a higher abundance of *Faecalibacterium prausnitzii* in older adults may be associated with a healthier gut microbiome and better overall health status 24, 25. Maintaining adequate levels of *Faecalibacterium* may help improve metabolic health, reduce inflammation, and promote overall healthy aging 26. Therefore, the observed reduction in these bacterial taxa may reflect age-related gut microbiota changes commonly seen in the elderly and could play a role in the development of HTN.

*Coprobacter* is another microbe in which we observed decreased abundance in HTN patients, and this result has been similarly observed in another study. Researchers have noted that *Coprobacter* is relatively more abundant in the normal blood pressure group compared to HTN patients 27. This discrepancy may suggest a certain association between blood pressure regulation and the variations in the microbial community. In our top 20 ASVs analysis, we observed a significant increase in *Tyzzerella* abundance among HTN patients. A prior study has reported a positive correlation between the abundance of *Tyzzerella* and systolic or diastolic blood pressure, suggesting a potential association with HTN 28. Additionally, research has indicated a link between *Tyzzerella* and the risk of cardiovascular disease (CVD), with higher abundance of *Tyzzerella* observed in individuals at high CVD risk 29, 30. This implies that reducing the abundance of* Tyzzerella* might be a strategy to lower the risk of HTN and cardiovascular disease occurrence.

We used Tax4Fun2 to predict the potential functional composition in HTN and found that in the HTN group, there was relatively higher activity or abundance of adenine modification enzymes and DNA methylases. These two enzymes are involved in epigenetic modifications, which may impact the pathogenesis, duration, and severity of HTN 31-33. On the contrary, in the HTN group, neutral ceramidase and Lys-Lys/Arg-Xaa endopeptidase are relatively inactive or less abundant. Ceramide is a biologically active lipid molecule that plays a crucial role in cell membrane composition and cellular signal transduction. Previous research has found elevated levels of ceramide in HTN individuals 34. Furthermore, studies have indicated that elevated ceramide levels are closely associated with cerebrovascular diseases, particularly stroke and small vessel disease 35. Endopeptidases can cleave peptide bonds within protein molecules, generating multiple active fragments that impact various physiological processes in the organism. Previous research has reported that under the catalytic action of endopeptidases, the conversion of angiotensin occurs, which subsequently regulates blood pressure 36.

In the HTN group, there are higher relative abundances in functional pathways such as Linoleic acid metabolism and the Renin-angiotensin system. Linoleic acid is a crucial polyunsaturated fatty acid and is classified as an omega-6 fatty acid, making it one of the essential fatty acids for the human body. Linoleic acid undergoes metabolic transformations in the human body, leading to the production of a series of biologically active substances that play a significant role in regulating blood pressure 37. The renin-angiotensin-aldosterone system (RAAS) is a regulatory mechanism within the endocrine system, associated with various diseases, including HTN, cardiovascular, and renal diseases. Excessive activity of the RAAS can lead to the development of HTN 38. Furthermore, recent research has reported an association between high-salt diets 39 and the composition of the gut microbiota 40 with the RAAS, subsequently impacting blood pressure. The alterations in the microbial community in specific functions and pathways may be associated with the development of HTN. However, further research is needed to validate these findings and to elucidate the precise role of the microbiota in HTN.

A primary limitation of this study is the modest sample size, potentially impacting the reliability and generalizability of our conclusions, particularly when extrapolating to the broader Taiwanese population beyond Chiayi City. Larger, longitudinal studies are essential to definitively validate causal associations across diverse regions of Taiwan. Even with this acknowledged limitation, our foundational data on *Faecalibacterium* provides a valuable pilot dataset for further investigation into the gut microbiome of HTN Taiwanese individuals within this region and beyond. This study analyzed the gut microbiota of elderly HTN patients in a specific region of Taiwan, with the primary findings being a decrease in *Faecalibacterium*. Previous studies from China 18, 41, 42 and Brazil 43 have reported an association between HTN and reduced *Faecalibacterium* abundance. This study is the first to document this change in the bacterial genus among elderly HTN patients in Taiwan, contributing valuable data from Taiwan to the global understanding of HTN-related gut microbiota alterations.

**Figure 5:**
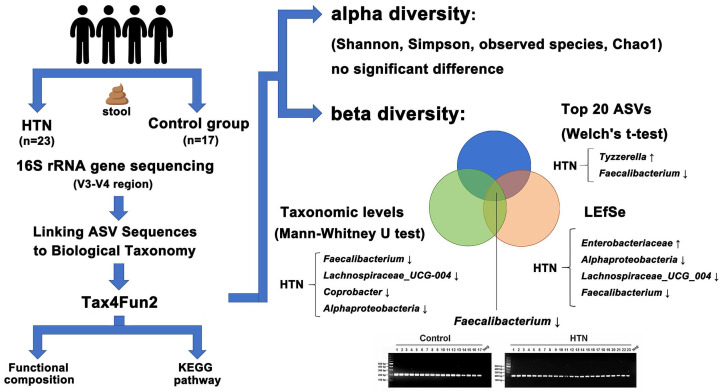
** Graphical summary of gut microbiota analysis in HTN and control groups.** Alpha diversity showed no significant differences, while beta diversity analysis revealed microbial shifts. In HTN, *Tyzzerella* increased, whereas *Faecalibacterium* decreased among the top 20 ASVs. Taxonomic analysis (Mann-Whitney U test) identified reductions in *Faecalibacterium*, *Lachnospiraceae_UCG-004*, *Coprobacter*, and *Alphaproteobacteria*. LEfSe highlighted increased *Enterobacteriaceae* and decreased *Alphaproteobacteria*, *Lachnospiraceae_UCG-004*, and *Faecalibacterium*. Functional predictions suggested metabolic alterations.

As illustrated in Figure 5, this study compared the gut microbiota composition between HTN patients and the control group, revealing a significant decrease in *Faecalibacterium*, *Lachnospiraceae_UCG-004*, and *Coprobacter*, along with an increase in *Tyzzerella*, *Lachnospiraceae_FCS020_group*, *Enterobacterales*, and *Enterobacteriaceae* in HTN individuals. These alterations may contribute to HTN by reducing butyrate production, exacerbating inflammation, and activating the RAAS pathway. Furthermore, insights into the potential pathogenic mechanisms underlying these microbial differences provide a foundation for the development of novel biomarkers and therapeutic targets.

## Supplementary Material

Supplementary figures.

## Figures and Tables

**Table 1:** Patient characteristics

Characteristics	Control group(n=17)	HTN group(n=23)	*p*-value
Ages (years) (Mean ± SEM)	72.59 ± 1.348	71.57 ± 1.538	*p* = 0.653
Sex, n (%)			*p* = 0.935
Male	13(76.5%)	17(73.9%)	
Female	4(23.5%)	6(26.1%)	
BMI (kg/m2) (Mean ± SEM)	22.75 ± 0.8170	26.19 ± 0.7438	*p* = 0.018
			
DM (Diabetes), n (%)	3(17.6%)	10(43.5%)	*p* = 0.035
HLP(Hyperlipidemia), n (%)	1(5.9%)	9(39.1%)	*p* = 0.002
PUD (Peptic ulcer disease), n (%)	4(23.5%)	1(4.3%)	*p* = 0.043
Smoking, n (%)	5(29.4%)	3(13.0%)	*p* = 0.258
Drinking, n (%)	4(23.5%)	1(4.3%)	*p* = 0.043
Cancer, n (%)	4(23.5%)	6(26.1%)	*p* = 0.559
BPH (Benign Prostatic Hyperplasia), n (%)	4(23.5%)	6(26.1%)	*p* = 0.559

## References

[1] Collaborators GDaI (2020). Global burden of 369 diseases and injuries in 204 countries and territories, 1990-2019: a systematic analysis for the Global Burden of Disease Study 2019. Lancet.

[2] He FJ, Tan M, Ma Y, MacGregor GA (2020). Salt Reduction to Prevent Hypertension and Cardiovascular Disease: JACC State-of-the-Art Review. J Am Coll Cardiol.

[3] Fernandes-Rosa FL, Boulkroun S, Fedlaoui B, Hureaux M, Travers-Allard S, Drossart T (2023). New advances in endocrine hypertension: from genes to biomarkers. Kidney Int.

[4] Zhao Q, Kelly TN, Li C, He J (2013). Progress and future aspects in genetics of human hypertension. Curr Hypertens Rep.

[5] Visconti A, Le Roy CI, Rosa F, Rossi N, Martin TC, Mohney RP (2019). Interplay between the human gut microbiome and host metabolism. Nat Commun.

[6] Gebrayel P, Nicco C, Al Khodor S, Bilinski J, Caselli E, Comelli EM (2022). Microbiota medicine: towards clinical revolution. J Transl Med.

[7] Yang Z, Wang Q, Liu Y, Wang L, Ge Z, Li Z (2023). Gut microbiota and hypertension: association, mechanisms and treatment. Clin Exp Hypertens.

[8] O'Donnell JA, Zheng T, Meric G, Marques FZ (2023). The gut microbiome and hypertension. Nat Rev Nephrol.

[9] Chen Z, Liang W, Liang J, Dou J, Guo F, Zhang D (2023). Probiotics: functional food ingredients with the potential to reduce hypertension. Front Cell Infect Microbiol.

[10] Evangelou E, Warren HR, Mosen-Ansorena D, Mifsud B, Pazoki R, Gao H (2018). Genetic analysis of over 1 million people identifies 535 new loci associated with blood pressure traits. Nat Genet.

[11] Avery EG, Bartolomaeus H, Maifeld A, Marko L, Wiig H, Wilck N (2021). The Gut Microbiome in Hypertension: Recent Advances and Future Perspectives. Circ Res.

[12] Durgan DJ, Ganesh BP, Cope JL, Ajami NJ, Phillips SC, Petrosino JF (2016). Role of the Gut Microbiome in Obstructive Sleep Apnea-Induced Hypertension. Hypertension.

[13] Marques FZ, Mackay CR, Kaye DM (2018). Beyond gut feelings: how the gut microbiota regulates blood pressure. Nat Rev Cardiol.

[14] Huart J, Leenders J, Taminiau B, Descy J, Saint-Remy A, Daube G (2019). Gut Microbiota and Fecal Levels of Short-Chain Fatty Acids Differ Upon 24-Hour Blood Pressure Levels in Men. Hypertension.

[15] Bartolomaeus H, Balogh A, Yakoub M, Homann S, Markó L, Höges S (2019). Short-Chain Fatty Acid Propionate Protects From Hypertensive Cardiovascular Damage. Circulation.

[16] Machiels K, Joossens M, Sabino J, De Preter V, Arijs I, Eeckhaut V (2014). A decrease of the butyrate-producing species Roseburia hominis and Faecalibacterium prausnitzii defines dysbiosis in patients with ulcerative colitis. Gut.

[17] Gomez-Arango LF, Barrett HL, McIntyre HD, Callaway LK, Morrison M, Dekker Nitert M (2016). Increased Systolic and Diastolic Blood Pressure Is Associated With Altered Gut Microbiota Composition and Butyrate Production in Early Pregnancy. Hypertension.

[18] Yan Q, Gu Y, Li X, Yang W, Jia L, Chen C (2017). Alterations of the Gut Microbiome in Hypertension. Front Cell Infect Microbiol.

[19] Calderón-Pérez L, Gosalbes MJ, Yuste S, Valls RM, Pedret A, Llauradó E (2020). Gut metagenomic and short chain fatty acids signature in hypertension: a cross-sectional study. Sci Rep.

[20] Zhang J, Song L, Wang Y, Liu C, Zhang L, Zhu S (2019). Beneficial effect of butyrate-producing Lachnospiraceae on stress-induced visceral hypersensitivity in rats. J Gastroenterol Hepatol.

[21] Tan C, Wu Q, Wang H, Gao X, Xu R, Cui Z (2021). Dysbiosis of Gut Microbiota and Short-Chain Fatty Acids in Acute Ischemic Stroke and the Subsequent Risk for Poor Functional Outcomes. JPEN J Parenter Enteral Nutr.

[22] Ferguson JF, Aden LA, Barbaro NR, Van Beusecum JP, Xiao L, Simmons AJ (2019). High dietary salt-induced dendritic cell activation underlies microbial dysbiosis-associated hypertension. JCI Insight.

[23] Jeffery IB, Lynch DB, O'Toole PW (2016). Composition and temporal stability of the gut microbiota in older persons. Isme j.

[24] Fart F, Rajan SK, Wall R, Rangel I, Ganda-Mall JP, Tingö L (2020). Differences in Gut Microbiome Composition between Senior Orienteering Athletes and Community-Dwelling Older Adults. Nutrients.

[25] Ghosh TS, Shanahan F, O'Toole PW (2022). The gut microbiome as a modulator of healthy ageing. Nat Rev Gastroenterol Hepatol.

[26] Claesson MJ, Jeffery IB, Conde S, Power SE, O'Connor EM, Cusack S (2012). Gut microbiota composition correlates with diet and health in the elderly. Nature.

[27] Dan X, Mushi Z, Baili W, Han L, Enqi W, Huanhu Z (2019). Differential Analysis of Hypertension-Associated Intestinal Microbiota. Int J Med Sci.

[28] Li H, Liu B, Song J, An Z, Zeng X, Li J (2019). Characteristics of Gut Microbiota in Patients with Hypertension and/or Hyperlipidemia: A Cross-Sectional Study on Rural Residents in Xinxiang County, Henan Province. Microorganisms.

[29] Kelly TN, Bazzano LA, Ajami NJ, He H, Zhao J, Petrosino JF (2016). Gut Microbiome Associates With Lifetime Cardiovascular Disease Risk Profile Among Bogalusa Heart Study Participants. Circ Res.

[30] Ascher S, Reinhardt C (2018). The gut microbiota: An emerging risk factor for cardiovascular and cerebrovascular disease. Eur J Immunol.

[31] Guo Y, Pei Y, Li K, Cui W, Zhang D (2020). DNA N(6)-methyladenine modification in hypertension. Aging (Albany NY).

[32] Liang M (2018). Epigenetic Mechanisms and Hypertension. Hypertension.

[33] Richard MA, Huan T, Ligthart S, Gondalia R, Jhun MA, Brody JA (2017). DNA Methylation Analysis Identifies Loci for Blood Pressure Regulation. Am J Hum Genet.

[34] Spijkers LJ, van den Akker RF, Janssen BJ, Debets JJ, De Mey JG, Stroes ES (2011). Hypertension is associated with marked alterations in sphingolipid biology: a potential role for ceramide. PLoS One.

[35] Yuan H, Zhu B, Li C, Zhao Z (2023). Ceramide in cerebrovascular diseases. Front Cell Neurosci.

[36] Jalil JE, Ocaranza MP, Oliveri C, Córdova S, Godoy I, Chamorro G (2004). Neutral endopeptidase and angiotensin I converting enzyme insertion/deletion gene polymorphism in humans. J Hum Hypertens.

[37] Hajihashemi P, Feizi A, Heidari Z, Haghighatdoost F (2023). Association of omega-6 polyunsaturated fatty acids with blood pressure: A systematic review and meta-analysis of observational studies. Crit Rev Food Sci Nutr.

[38] Nwia SM, Leite APO, Li XC, Zhuo JL (2023). Sex differences in the renin-angiotensin-aldosterone system and its roles in hypertension, cardiovascular, and kidney diseases. Front Cardiovasc Med.

[39] Ertuglu LA, Pitzer Mutchler A, Elijovich F, Laffer CL, Sheng Q, Wanjalla CN (2023). Regulation of human salt-sensitivite hypertension by myeloid cell renin-angiotensin-aldosterone system. Front Physiol.

[40] Mizoguchi R, Karashima S, Miyajima Y, Ogura K, Kometani M, Aono D (2023). Impact of gut microbiome on the renin-aldosterone system: Shika-machi Super Preventive Health Examination results. Hypertens Res.

[41] Li J, Zhao F, Wang Y, Chen J, Tao J, Tian G (2017). Gut microbiota dysbiosis contributes to the development of hypertension. Microbiome.

[42] Mushtaq N, Hussain S, Zhang S, Yuan L, Li H, Ullah S (2019). Molecular characterization of alterations in the intestinal microbiota of patients with grade 3 hypertension. Int J Mol Med.

[43] Silveira-Nunes G, Durso DF Jr L, Cunha EHM, Maioli TU, Vieira AT (2020). Hypertension Is Associated With Intestinal Microbiota Dysbiosis and Inflammation in a Brazilian Population. Front Pharmacol.

