# Supplementary material for: Characterizing the gut microbiota composition in Taiwanese hypertensive patients using 16S rRNA sequencing analysis
Source: Int J Med Sci. 2025 Apr 28;22(10):2460–9. doi: 10.7150/ijms.109340 (PMC12080567; doi:10.7150/ijms.109340)
Supplement: Supplementary file 1 — Supplementary figures. [file ijmsv22p2460s1.pdf]

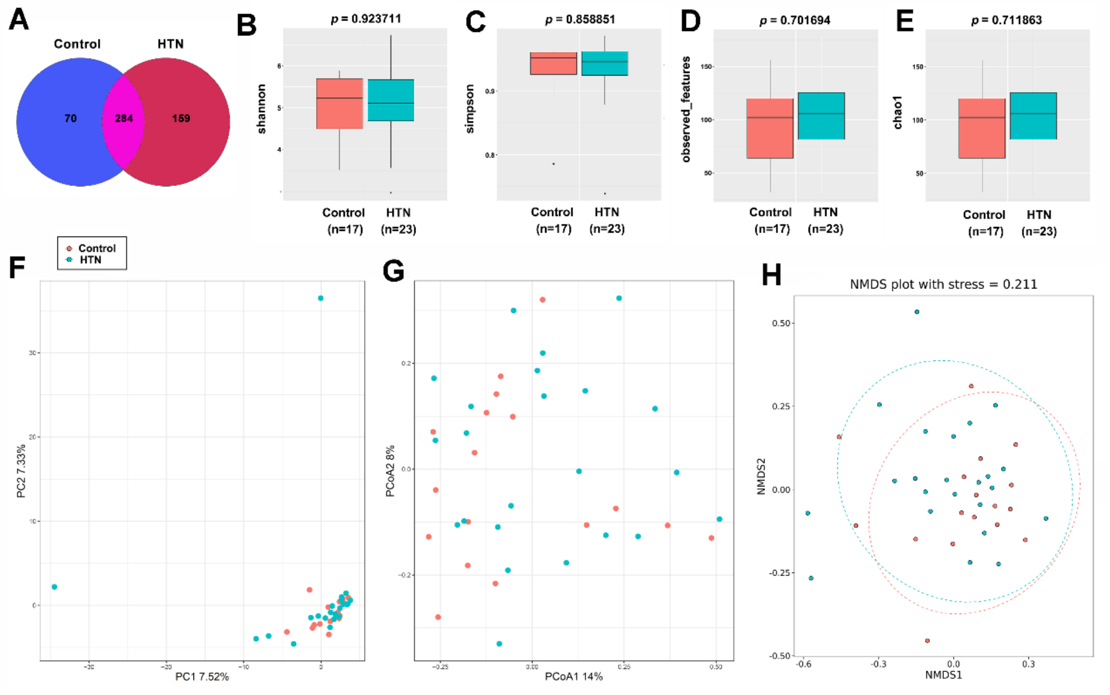

Supplementary Figure 1. Diversity analysis of the intestinal microbiota in patients with HTN and the control group. (A) The Venn diagram illustrates the number of shared and unique ASVs identified in the fecal microbiota between the two groups (HTN versus control). (B-E) The histogram illustrates the comparison of intra-sample diversity using the α-diversity index, including Shannon (B), Simpson (C), observed_features (D), and Chao1 (E), to uncover the diversity differences of the gut microbiota within the two groups. (F-H) The scatter plot of results illustrates the comparison between the HTN group and the control group, where each data point represents a sample. Principal components analysis (PCA) plot depicts the relative positions of data points on PC1 and PC2, representing the main patterns and relationships within the dataset as linear combinations of the original variables (F). Principal coordinates analysis (PCoA) plot depicts inter-sample similarity and dissimilarity using a (dis)similarity matrix as input (G). Non-metric multidimensional scaling (NMDS) analysis plot demonstrates dissimilarity between gut microbiota communities of HTN group and control group, utilizing rank metrics to reduce sensitivity and highlight differences. NMDS plot (stress = 0.211) reveals distinct differences in gut microbiota community between HTN and control groups (H).

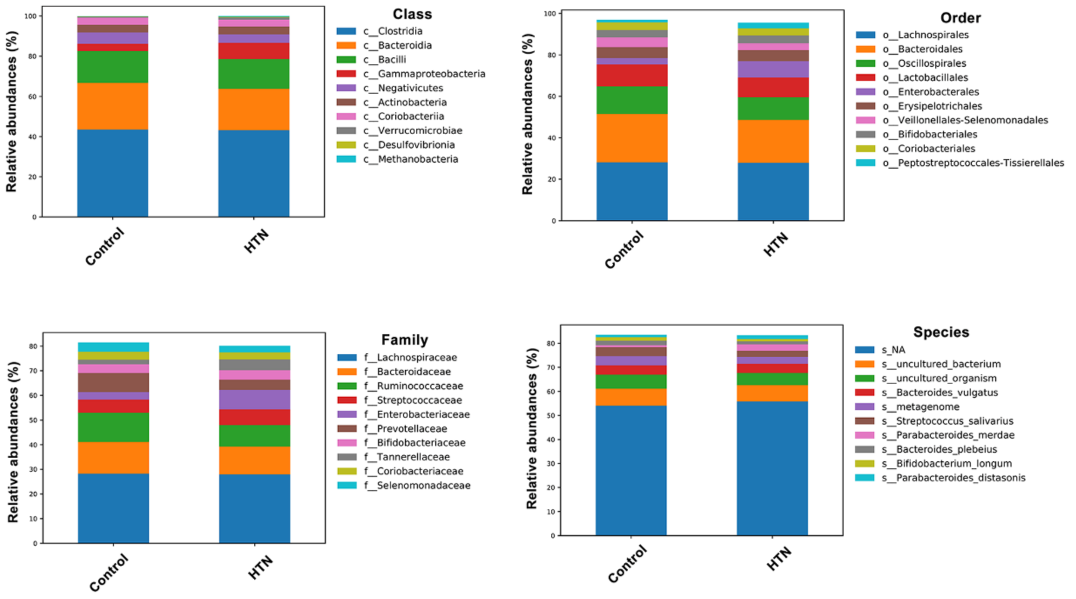

Supplementary Figure 2. Comparison of relative abundance of gut microbiota between the HTN and control groups across different taxonomic levels (Class, Order, Family, Species).
